# Ontology-Oriented Diagnostic System for Traditional Chinese Medicine Based on Relation Refinement

**DOI:** 10.1155/2013/317803

**Published:** 2013-02-19

**Authors:** Peiqin Gu, Huajun Chen, Tong Yu

**Affiliations:** ^1^College of Computer Science, Zhejiang University, Hangzhou 310027, China; ^2^China Academy of Chinese Medical Sciences, Beijing 100700, China

## Abstract

Although Chinese medicine treatments have become popular recently, the complicated Chinese medical knowledge has made it difficult to be applied in computer-aided diagnostics. The ability to model and use the knowledge becomes an important issue. In this paper, we define the diagnosis in Traditional Chinese Medicine (TCM) as discovering the fuzzy relations between symptoms and syndromes. An Ontology-oriented Diagnosis System (ODS) is created to address the knowledge-based diagnosis based on a well-defined ontology of syndromes. The ontology transforms the implicit relationships among syndromes into a machine-interpretable model. The clinical data used for feature selection is collected from a national TCM research institute in China, which serves as a training source for syndrome differentiation. The ODS analyzes the clinical cases to obtain a statistical mapping relation between each syndrome and associated symptom set, before rechecking the completeness of related symptoms via ontology refinement. Our diagnostic system provides an online web interface to interact with users, so that users can perform self-diagnosis. We tested 12 common clinical cases on the diagnosis system, and it turned out that, given the agree metric, the system achieved better diagnostic accuracy compared to nonontology method—92% of the results fit perfectly with the experts' expectations.

## 1. Introduction

 Applying mathematical models and information technologies to medical intelligence has long been a hot spot in the academic research domains and real-life health care applications. Plenty of the efforts in this field allow researchers and medical practitioners to identify required information more efficiently, discover new substances or relationships, and integrate different sources of information more easily.

Traditional Chinese Medicine, known as TCM, that is an ancient and unique branch of medical science, which probably covers broad range of practices such as herb medicine, acupuncture, attracts much attention on how to defeat the inconsistency of therapeutic patterns and vagueness of medical terms in TCM in order to improve the user experience of TCM diagnosis.

The basic theories of TCM are based on the ancient philosophy of holistic understanding of the universe and the human body, which are commonly associated with the flow of Qi and the balancing of complementary opposites, such as Yin and Yang, or five elements. Based on the interacting forces in the human body, the forms of many pathological conditions in TCM usually differ from that of identifiable diseases in terminology of the Western medicine. “*Syndrome*” (also called pattern) refers to a pattern of disharmony or functional disturbance within the functional entities that the TCM model of the body is composed of. A “*disease*” in TCM refers to disease entity or disease category, focusing on the macroscopic classification of specific manifestations. In TCM diagnosis, the therapy is determined mainly according to the pattern rather than the disease. Two patients with the same disease but different patterns will receive different therapy, vice versa patients with similar patterns might receive similar therapy even if their diseases are different. Hence, the concept of “syndrome differentiation” is of great importance in deciding the diagnosis for patients. But the difficulty of differentiating syndromes is rooted in the unclear definition of the range of syndromes, which means the determination of specific syndromes, the similarity among syndrome patterns, and these information are usually hidden in the literature texts or the doctors' experience.

In this paper, we propose a simple but useful diagnostic system based on a domain ontology proposed by professional TCM experts, which is supposed to formalize the hidden knowledge and be imported into the diagnostic process. Similar to other TCM diagnostic systems, this framework basically includes two important components: *clinical database of patient data* and *knowledge base of patterns or classifiers*. 10,000 high-quality cases were collected, involving 5 types of diseases, 732 types of syndromes, and 3,519 symptoms, from a big clinical database of China Academy of Chinese Medicine Sciences (CATCM), a national research institute in Beijing. In the feature extraction phase, symptoms related to the same syndrome or same disease from different clinical cases are calculated by an adaptive intersection algorithm to generate a minimum symptom set. The goal of this step is in fact feature selection in a reductive way, in which some key symptoms are the features in order to identify a syndrome or a disease. However, there are still three problems to solve: *concept subsumption*, *synonym*, *inclusion* relations among syndromes. The *Ontology-oriented Diagnostic System (ODS)* address these problems by applying *ontology representation* as one of the techniques of semantic technologies, to systematize the important but usually hidden knowledge of syndrome relationships. It captures all the subtle relationships among syndromes as a terminology base and stores it in an ontology file. The benefit of building specific ontology is to enable the standardized terminology description of scientific domain and smooth data interchange on the web. In ODS, the ontology of syndromes is used to rectify the initial diagnosis results derived from the predetermination of syndrome-symptom, disease-symptom relations by rechecking the associated syndromes, also called a *relation refinement* process. A Web-based GUI is especially designed for this system to allow user-friendly instant diagnosis without human intervention. To evaluate the usefulness of the system, we invited TCM experts to validate the accuracy of our system by scoring each diagnostic result, and the feedback turned out promising.

The major works in this paper are the following.

- Use clinical data as a feature selection source, and propose a new computation model for initial decision making of TCM diagnosis. The aim is to find the most closely related set of symptoms for each occurred syndrome or disease entity, which can be regarded as a statistical mapping table.
- With the broad use of biomedical ontologies, a simple ontology is designed for describing the interrelationships between syndromes, as an attempt to define the range and overlap of syndromes. The inheritance and subsumption will be used to distinguish between them.
- Based on the expertise-dependent characteristic of TCM diagnosis, an agree metric is proposed to evaluate the efficiency of our diagnostic system, which is actually ranking of the diagnostic results. The scores suggested that ODS shows certain potential in making diagnostic decisions.

The rest of the paper is organized as follows.  Section 2 outlines related research in the area.  Section 3 gives a clear formalization of the medical diagnosis problem.  Section 4 gives a detailed introduction of the kinds of relationships we built in the syndrome ontology. In Section 5, we demonstrate the minimum set extraction algorithm applied on the clinical database. In Section 6, after constructing mapping relations between the syndromes and symptoms based on the clinical records, relation refinement based on ontology hierarchy will be applied on the initial diagnostic result to finalize it.  Section 7 gives a demonstration of a web-based diagnostic platform and evaluates the accuracy, performance, and scalability of the diagnostic capability of the system. Finally, we conclude and discuss the future work in Section 8.

## 2. Related Work

 In the domain of medical diagnosis systems, a multitude of approaches exist, including various algorithmic techniques for intelligent automatic diagnosis that has been used to solve practical diagnostic problems in TCM, as well as in Western medicine, such as Bayesian networks, ontology engineering, and rule-based inference.

The design of automatic TCM diagnostic systems usually has to deal with the unstructured clinical information and vague description of domain knowledge. Bayesian classifiers such as Naïve Bayesian classifier are the most popular methods that have been applied in TCM diagnostics to the learning and classification of disease and syndrome based on symptoms. Moreover, Bayesian network algorithms are used to construct knowledge bases from clinical data [1]. A self-learning system for diagnosis in TCM [2] constructed a knowledge base by applying an improved hybrid Bayesian network learning algorithm with data mining techniques. By performing Naïve-Bayes classifiers with a score-based strategy for feature selection and a method for mining constrained association rules, the diagnostic results of the system turned out encouraging.

In recent years, to facilitate biomedical research among communities, various ontologies and knowledge bases have been developed [3], for example the *Gene Ontology (GO)*, *UMLS* [4], and *SNOMED RT* [5], to provide the capability of knowledge management, knowledge sharing. Compared to Bayesian methods, the semantic web technologies benefit the biomedical domain by providing a conceptualisation of the domain with the means of specifying the concepts and the relationships into a standardized form. When being extended to medical domains, semantic technologies can reveal machine-readable latent relationships within information where the homogeneity of terminology is particularly critical. Knowledge-based systems for medical diagnosis such as [6] build a formal ontology to manage medical information and suggest that the use of semantic technologies could improve the accuracy of medical diagnosis. ODDIN, an ontology-driven medical diagnosis system [7], uses a number of knowledge-based technologies to solve the problems of ambiguity which is very common in medical diagnosis, such as ontologies representing specific structure information and probabilistic statistical refinements. The semantic ontology support in ODDIN benefits the diagnostics with the reasoning and inference capabilities offered by ontologies.

In the field of TCM, the emergence of knowledge-based diagnostic systems might improve the understanding of medical diagnostic process which is based on philosophical theories. A knowledge-based Chinese Medical Diagnostic System (CMDS) [8] extracted an integrated medical ontology with described domain knowledge and provided a standardized understanding of digestive disease diagnosis. By constructing a hierarchical ontology and association rules as classifiers, the system facilitated the acquisition, verification, and maintenance of knowledge by both human and machines. This prototype of CMDS can diagnose about 50 types of diseases by using over 500 rules and 600 images for various diseases. For those who is looking for more work on extracting ontology statements to build a robust specialized knowledge base for TCM, [9] is a good reference work that have been done by Zhejiang University, which includes more than 10,000 classes and about 80,000 instances.

According to a recent survey on computational methods for TCM [10], although there have been several TCM approaches employing different ontologies, standardized ontology seems absolutely necessary. In order to get richer and more adequate diagnostic results in TCM diagnostic systems, information from domain ontologies should be well used.

## 3. Problem Formulation

 A medical diagnosis problem can be formed as a probabilistic relation *R* ⊂ *S* × *D* between a group of symptoms *S* and a diagnosis *D*. Let *S* = {*s*_1_, *s*_2_,…, *s*_*m*_} be the set of symptoms to be queried, in which *m* is the cardinality of *S*, namely, the number of symptoms. Each *s*_*i*_ ∈ *S*, 1 ≤ *i* ≤ *m* is a professional description of a pathological symptom, such as *cough* and *fever*. The diagnostic result *D* = *C* ⊕ *Q* is an integrated answer of some disease and at least one syndrome, meaning that the patient catches a disease named *C* with a collection of syndromes *Q*.

The difficulties in determining this relation *R* between *S* and *D* lie on the obscure descriptions of each clinic case and uncertain (expertise-dependent) differentiation of syndromes. As an outsider of medical science, we are suggested by the medical experts that a relation table *P* should be defined in advance, in which there are two kinds of relations *R*_1_ and *R*_2_. *R*_1_ represents the correspondence between a specific *disease* and a set of *symptoms* and *R*_2_ represents the correspondence between a specific *syndrome* between a set of *symptoms*, and a table of these correspondence relations form a feature selection source for medical diagnosis. For example, we could obtain a minimum associated set of symptoms for each disease or syndrome such as {*Heat* → (*heat*, *dizzy*)} by analyzing prior clinical data {*Heat* → (*heat*, *dizzy*, *redface*)},{*Heat* → (*heat*, *dizzy*, *sore* *throat*)}, which is formatted as (disease *C* or syndrome *Q*, related symptoms *S*). The process of clustering and filtering data is a variant of set intersection problem. We put our considerations on the *performance* and *efficiency* of the algorithm.

In order to rectify the data biases of clinical data and facilitate knowledge-based diagnosis, we define a knowledge base *B* which takes the role of the conceptual model. In the procedure of automatic diagnosis, the user-queried symptoms *S* are compared with the relation *R* generated from the preprocessed relation table *P* and the knowledge base *B*. By calculating the similarity, the diagnostic result *D* will be given as the combined answer of a disease *C* and a group of syndromes *Q*.

The process of Chinese medical diagnosis can be concluded as an expertise-dependent case search based on observed pathological patterns, where expertise is consisted of practical experiences and theoretical principles. In this paper, we integrate both practical experiences and theoretical principles together to construct a complete computation model for reliable medical diagnosis.

## 4. Ontology Modelling for TCM Syndromes

 In TCM, syndrome refers to the association of several clinically recognizable features, usually identified by a group of symptoms that collectively indicate or characterize a disease, psychological disorder or other abnormal condition. More and more medical researchers recognize that the combination of disease diagnosis in biomedicine and pattern differentiation in TCM is essential for the clinical practice, and it has been a common practice model in China, since it will produce better clinical effects. Syndrome differentiation will help improve the clinical efficacy in clinical practice since it further specifies the indication with TCM classification. Syndrome differentiation is mainly based on symptoms, including tong appearance, pulse palpation, and patient's mental state, which aims to classify the symptoms into specific groups. However, the syndromes found in TCM are not totally independent, instead there are certain connections among them, for example, subsumption, equivalence, and disjointness.

Semantic web technologies are currently widely used in the biomedical domain [11], capturing, structuring, retaining, and reusing information to develop an understanding of the whole system (e.g., genome, pathway), and subsequently to convey this information meaningfully to other information systems. One of the methods to represent biomedical knowledge is to build a comprehensive terminology ontology, sometimes specific to the applications at first, but may be easily adapt to further integration. The basic idea of an ontology is to construct abstract knowledge by resource triples 〈*s*, *p*, *o*〉, which states that a relation denoted by *p* exists between subject *s* and object *o*. For example, we could declare a statement that 〈*Insomnia*, *causes*, *easy* *to* *wake*〉, in which *Insomnia* is the subject disease, *easy to wake* is a pathological symptom *caused* by the disease. The representation languages used to build ontology is proposed by W3C consortium, such as Resource Description Framework (RDF) [12] and Web Ontology Language (OWL) [13].

**Figure 1:**
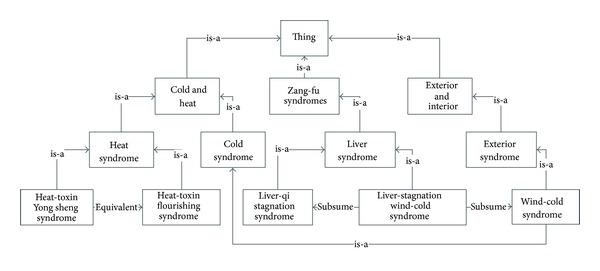
A portion of the hierarchy of syndrome ontology model.

An ontology of syndromes called *SynOnt* is designed to structure the interrelationships among TCM syndromes, which should also be available for further extension and integration (file published at https://www.github.com/astergu/TCM_Link/blob/master/syndrome.owl/). Generally, an ontology of high quality is developed from curated knowledge maintained by domain experts [14]. In order to extract a set of concise statements as a reliable knowledge base, one research scientist in CATCM was invited to build the ontology manually in 2009 for a month. We did a systematic bibliographic analysis on three widely acknowledged teaching materials “Basic Theories of Chinese Medicine” [15], “Therapeutic Science in Chinese Medicine” [16], and “Inner Medicine of Chinese Medicine” [17]. The ontology was developed using the open source ontology editor Protégé, by first creating top categorical concepts and corresponding subconcepts, then for related syndromes, connect them with property relations. The standard naming of the syndromes and the relations between them were fully discussed and modelled as semantic classes and properties. As shown in Figure 1, *SynOnt* ontology deals with three major problems related to syndrome differentiation, equivalence (equivalent), inheritance (is-a), and subsumption (subsume). 

### 4.1. Equivalent Syndromes

 There are some circumstances that exist multiple names for one identical thing, especially happen a lot in medicine, for example, the plant *Rheum palmatum* is also called *Fire ginseng*. This kind of knowledge appears as common sense for domain experts, but rather difficult for computers to know and understand. Identifying obvious similar references help merge different sources of knowledge together and reduce the cost of data mining and knowledge discovery.

The built-in OWL property *owl:equivalentClass* links a class description to another class description, which indicates that the two URI references actually refer to the same concept; namely, they have the same class extension. The *owl:equivalentClass* statements are often used in definition mapping between ontologies. For an individual syndrome class such as *“deficient cold syndrome,”* we could state that the following three URI references actually refer to the same kind of syndrome:

<owl:Class rdf:about = "Deficient cold syndrome">

<owl:equivalentClass rdf:resource = "Cold syndrome">

<owl:equivalentClass rdf:resource = "Yang deficient syndrome">

</owl:Class>

By that mean, we say *deficient cold syndrome* is the same as both *yang deficiecy syndrome* and *cold syndrome*, which can be easily imported to other data sources with inference tools.

### 4.2. Syndrome Inheritance

 The inheritance relationship between syndrome classes is defined in the categorical hierarchy of syndromes in the form of RDF triple 〈*C*_1_ *rdfs* : *subClassOfC*_2_〉. This relationship shows the affiliation relation between *C*_1_ and *C*_2_. If the class description *C*_1_ is defined as a subclass of class description *C*_2_, then the set of individuals in the class extension of *C*_1_ should be a subset of the set of individuals in the class extension of *C*_2_. This class axiom declares a subclass relation between two OWL classes that are described through their names.

The inheritance relationships between syndromes shows the categorical information in TCM diagnostics. Generally, the type of syndromes are divided into four major categories: *heat and cold*, *zang-fu*, *deficiency and excess* and *exterior and interior*, whose classification reflects the pathological deviation of syndromes. For a syndrome associated with a set of symptoms, we regard the set of symptoms as the extension of the specific syndrome. According to the diagnostic principle, the symptoms related to the subclass syndrome inherits the symptoms related to the superclass syndrome.

### 4.3. Syndrome Subsumption

 Based on the understanding of TCM therapeutic theories, we found that different resources of syndromes could not only be duplicated by name, but also associated with each other by relational algebra. For example, the syndrome *deficiency of lung-yin and kidney-yin* actually means the abnormal conditions of yin deficiency happen simultaneously both in lung and kidney, not varying much from the composition of *lung-yin deficiency* and *kidney-yin deficiency*.

We define a property relation *tcm:subsume* which extends the meaning of built-in property *owl:unionOf*. The *owl:unionOf* property links a class to a list of class descriptions. An *owl:unionOf* statement describes an anonymous class for which the class extension contains those individuals that occur in at least one of the class extensions of the class descriptions in the list. However, the inclusion relation *tcm:subsume* defined by us assigns the union of symptoms associated with a collection of individual syndromes, with a “or” relation.

<owl:Class rdf:about = "Qi stagnation and blood stasis">

<owl:equivalentClass>

<owl:Restriction>

<owl:onProperty rdf:resource = "subsume"/>

<owl:someValuesFrom rdf:resource = "Qi stagnation syndrome"/>

</owl:Restriction>

</owl:equivalentClass>

<owl:equivalentClass>

<owl:Restriction>

<owl:onProperty rdf:resource = "subsume">

<owl:someValuesFrom rdf:resource = "Blood stasis syndrome">  </owl:Restriction>

</owl:equivalentClass>

<rdfs:subClassOf rdf:resource = "Excess syndrome">

</owl:Class>

If it appears that *a tcm:subsume b* in user-friendly Manchester syntax, then it means syndrome *a* captures all the symptoms when syndrome *b* occurs, but not vice versa. If *a tcm:subsume b* and *a tcm:subsume c*, then *a* links to all the symptoms related to both *b* and *c*.

## 5. Syndrome-Symptom Relation Extraction from Clinical Data

 We propose a feature set extraction method based on a revised adaptive set intersection algorithm to preprocess the rows of clinical data. In fact, in the three-column design of the clinical database, *C* stands for the column of disease, *Q* for syndrome and *P* for symptoms. In general, disease in TCM is perceived as a category to distinguish the disharmony (or imbalance) in the functions or interactions of yin, yang, qi, xue, zang-fu, and meridians and the interaction between the human body and the environment. In clinical practice, the identified pattern (syndrome) usually involves differentiation of occurred symptoms. Each row represents a clinical statement that once a patient caught all the symptoms as listed in *P*, then he or she was diagnosed to have the disease named *C* and the syndrome named *Q*. These clinical data are thought to be trustworthy and of high quality; since they are extracted from another journal paper database of CATCM, the data of which are collected from top TCM journals, such as “*Zhong Hua Zhong Yi Yao Za Zhi,*” “*Chinese Journal of Integrative Medicine*”. We informally express each row as an expression *C* ⊕ *Q* = *S*(*s*_1_, *s*_2_,…, *s*_*p*_) to describe the problem more easily. The same disease or the same syndrome usually occurs in more than one clinical cases. The following expressions demonstrate the possible scenarios referring to the clinical cases:

(1) $$\begin{array}{c} C_{1}⊕Q=S_{1}\left(s_{1},s_{2},\ldots ,s_{p}\right),\\ C_{2}⊕Q=S_{2}\left(s_{1}^{\prime },s_{2}^{\prime },\ldots ,s_{q}^{\prime }\right), \end{array}$$

(2) $$\begin{array}{c} C⊕Q_{1}=S_{1}\left(s_{1},s_{2},\ldots ,s_{p}\right),\\ C⊕Q_{2}=S_{2}\left(s_{1}^{\prime },s_{2}^{\prime },\ldots ,s_{q}^{\prime }\right). \end{array}$$

In TCM diagnosis, though it is difficult to build a complete model to understand the complicated theory, but it is acknowledged that the emergence of pathological patterns (such as syndromes, disease, and symptoms) shows certain laws. The frequency of the appearance of the same symptom shows the strength of connection to syndromes or diseases. The more the symptom appears in the disease or syndrome related expressions, the firmer the connection is. Thus, we transform the frequency calculation problem of symptoms to a set intersection problem.

The intersection problem in processing the clinical data can be defined as finding the relatively small but compact set of symptoms for each disease and syndrome. For the expressions (1), we should conclude that the syndrome *Q* is coherently related to the mixed set of symptoms *S* = *S*_1_⋂*S*_2_; similarly, the disease *C* can be mapped to the set of symptoms *S* = *S*_3_⋂*S*_4_ concluded from the expressions (2). It is obvious that the elements found are the intersection of both sets. These expressions are collected and formalized directly from each row of clinical database, and the most relevant symptoms are concluded with the intersected sets.

Consider the problem of computing the intersection of *k* sets of various size, and the value of *k* might varies a lot for different test cases (different syndromes or diseases). In 2000, Demaine et al. [18] introduced a new intersection algorithm, named *Adaptive*, which intersects all the sets in parallel so as to compute the intersection in time proportional to the shortest proof of the result set. They studied that in the context of Internet information queries and text database systems. If queries are composed of words, and for each keyword a sorted set of references to entries in the database is precomputed, then the set of data entries in the database matching all the keywords of a query is the intersection of the sorted sets corresponding to each keyword. After that, Jérémy have done an experimental investigation [19] of set intersection algorithms and extend the optimal algorithm to the *t-threshold problem* [20], which consists in finding the elements which consists in finding the elements which are in at least *t* of the *k* sets.

**Figure 2:**
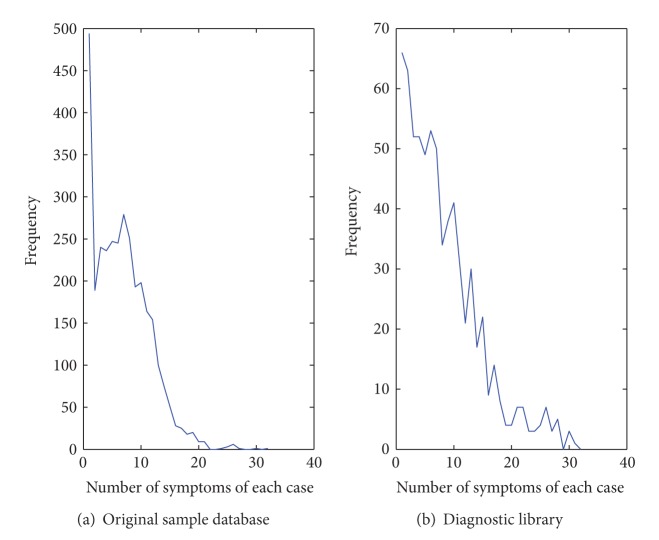
Number of clinical cases by length.

Although in general case, sets *S* = {*S*_1_, *S*_2_,…, *S*_*n*_} are arbitrary, an important case is when each set *S*_*n*_ is already in order. In this case, the performance of set intersection algorithm can be enhanced. However, this is not optimal for all possible cases. In fact, if *m* is small, it is better to do *m* binary searches obtaining an *O*(*m*log⁡*n*) algorithm [21]. When *m* and *n* are both large, it is necessary to look up most of the elements as effective as possible. According to [20], any algorithm for the intersection problem must certify that the output is correct: first, it must certify that all the elements of the output are indeed elements of all the sets; second, it must certify that no element of the intersection has been omitted by exhibiting some inequalities which imply that there can be on other element in the intersection. Therefore, they prove that any deterministic algorithm for intersection problem must take at least *Ω*(*δ*∑_*i*_log⁡(*n*_*i*_/*δ*)). Here, *δ* is defined as the difficulty of an instance. Given *k* ordered sets *A*_1_, *A*_2_,…, *A*_*k*_ of sizes *n*_1_, *n*_2_,…, *n*_*k*_, the variant *t*-threshold problem consists in computing the set of elements which are present in at least *t* of *k* sets. In particular, *t* varies from the magnitude of *k*, namely, the cardinality of the input sets. We applied an altered adaptive intersection algorithm (Algorithm 1) based on *t*-threshold [20] to construct a feature selection library. We simplify the result of small data set and eliminate the doubling search process for unnecessary redundance. The content of the generated diagnostic library is a collection of expressions in the form of *D* ≈ *S*, in that *D* represents a kind of disease or syndrome, and *S* represents a set of most relevant symptoms. Usually the clinical data is incomplete and noisy, which may causes inadequate mining results. For example, the number of symptoms in each case varies over a wide range, from to 1 to 32, as shown in Figure 2, and the average quantity of symptoms for each row in the sample database is 6.787. After the process of data preprocessing, we generate 705 *D* ≈ *S* mapping relations with an average length of 8.21 of symptoms for each case. It is obvious that the length of symptoms becomes more well distributed, with less frequency near 0, more frequency near the center of the range. 

Through the set intersection calculation of original clinical records, each syndrome or disease is associated with a relatively minimum set of symptoms. The reason why both syndromes and diseases matter to the TCM diagnosis is that they will both be considered in the process of clinical diagnosis, and treatments will be taken under the classification results.

## 6. United System of Syndrome Differentiation

### 6.1. Ontology-Based Relation Refinement

 To overcome the heterogeneity of information sources of different domains and attribute volumes, ontologies based on conceptual graphs are integrated to represent component information, such as the field labels that contain the same kind of information from different databases [22]. The use of ontologies for the explication of implicit and hidden knowledge is a possible approach to overcome the problem of semantic heterogeneity, which eliminates the inconsistency of information resources and distributed information bases.

Semantic heterogeneity considers the content of an information item and its intended meaning. According to [23], there are three main causes for semantic heterogeneity: *confounding conflicts*, *scaling conflicts*, and *naming conflicts*. Naming conflicts occur when naming schemes of information differ significantly, such as homonyms and synonyms.

With respect to the integration of data sources, ontologies can be used for the identification and association of semantically corresponding information concepts. We built a single ontology as one global ontology providing a shared vocabulary for the specification of the semantics. All information sources are related to one global ontology. As mentioned in Section 2, we defined an *equivalent class* with no restriction as a synonym for syndromes, a *subClassOf* subsumption relation as a categorical definition, and a set of *restrictions* with object property relation as a customized relation “subsume.” The capability of using ontology will be enhanced by perform inference on it and generating new facts. 

**Figure 3:**
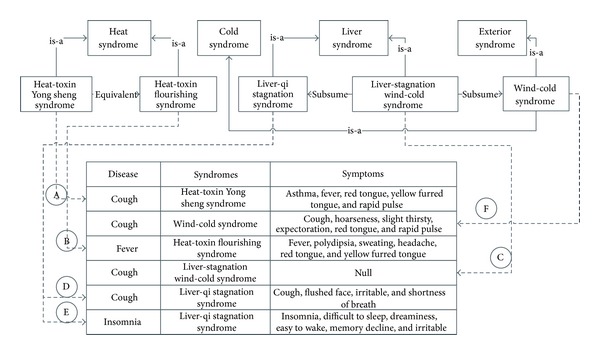
The integration of TCM domain knowledge and the clinical cases in the database.

 The integration process between ontology and database is demonstrated in Figure 3. In this figure, the hierarchy of syndromes consists the most important theoretical base for medical diagnosis. Relations between syndromes defined in ontology are integrated with rows of the clinical database in a directed way. We use strict text matching strategy to map the ontological definitions to database columns, specifically the “syndromes” column. We propose four strategies or steps for data integration after the first step of data preprocessing in Section 5; here *S* stands for the input set of symptoms, and *T* stands for currently matching symptom set.

1. *S*⊃*T*: the syndrome set *T* only matches part of the symptoms *S*, it means that the input syndromes may match some other syndrome with higher similarity or match the syndrome which *“subsume”* the syndrome *T* (*A tcm:subsume syndrome(T)*).
2. *S* ⊂ *T*: the symptom set *T* is much larger that the input set *S*, which means a smaller set may be more perfect, then we should consider the super classes of *syndrome(T)* (*syndrome(T) *⊃* superclass(syndrome(T))*).
3. *S* = *T*: the symptom set *S* matches perfectly with *S*, *syndrome(T)* or its equivalent syndrome should be a perfect answer.
4. *S*∩*T*: the input symptom set *S* has intersection with the possible symptom set *T* with a degree of matching above certain threshold, the unmatched part of input sets are recalculated for most relevant local result. The final result is consisted of the local results as combined syndromes. It is quite common in TCM that syndromes can happen together with one disease.

Here, *syndrome(T)* stands for the specific syndrome relates to the symptom set *T*. Instead of iteratively calculating the corresponding symptoms for each syndrome, we would rather postpone the integration of metaknowledge and databases, until the actual implementation of query answering.

### 6.2. Fuzzy Diagnosis

 When a patient comes, not knowing what kind of disease he or she has, must summarize his or her symptoms, including external appearance such as red face, yellow tongue fur, and detected symptoms including string pulse. When these symptoms are submitted to the ODS, they will be compared to the symptom-set list with similarity match algorithm to find the most similar set of disease *A* and syndrome *B*. After this, the ontology will be involved to make relation refinement according to the hierarchical information such as equivalence, subsumption, inheritance. The symptom set of the closely related syndromes of *B* will be recalculated. At last, the system will give the most relevant syndrome(s) and disease. In general, the main goal is to identify the most relevant diagnostic result (including disease and syndromes) for an input of symptoms. Due to the randomness of user-input symptoms, the input should be carefully matched with technical terms in the processed clinical database by measuring similarity.

Basically, since we are not ready for semantic text match, the measure of symptom set similarity of the system is based upon simple text match by identifying the long common subsequence [24]. The longest common subsequence measures the similarity letter by letter, which ranges from 0 to 1. The information of *weight* tells the ratio of matched string sequence over the longer one of the two pieces of texts. Frequency of full-text match (when *weight* equals to 1) is counted, and we multiply the sum of *weight* for each word by the frequency, then dividing the size of the symptom list (number of symptoms in the list). The similarity *sim* between input symptoms and each specific group of symptoms in the table *P* is a float number obtained during each comparison. The input set of symptoms is matched with each clinical case for each kind of diseases and syndromes. Both the disease and the syndrome of the final answer will be separately found out once the cases with the largest similarity pop out one has.

(3) $$\begin{array}{c} \text{sim}\left[\text{input},\text{list}\right]=\frac{\text{num}\left(\text{weight}=1\right)\times \sum \text{weight}}{\;\text{list}\cdot \text{size}\left(\;\right)},\\ \text{weight}=\frac{\text{length}\left(\text{lcs}\right)}{\text{length}\left(\max\left(a,b\right)\right)}. \end{array}$$

The process of obtaining diagnostic results is actually a process of sorting and filtering. An array of input symptoms *I* will be compared to each record of list *L* in the table *P* based on (3), in order to find an initial diagnostic result. The *fuzzy* here means that we assign a weighted value for both word matching and set matching. *Fuzzy word matching* inherited from basic text matching method defines a truth value that ranges in degree between 0 and 1, which measures the similarity between input words (user input) and technical terms (in the database). *Fuzzy set matching* takes both the sum of weights and the number of symptoms into account. We do not consider the fuzziness of descriptions in symptoms, such as a little pain, since it is another research problem referring to fuzzy logic. Several researches have been done to apply the fuzzy logic proposed by Zadeh [25] in 1965 into medical diagnosis [26].

## 7. Results and Validation

 Based on the methods we proposed, we implemented an interactive medical diagnostic platform deployed on web browser. Online diagnostic system can provide users with real-time clinical suggestions rather than actual medical diagnosis due to the lack of knowledge and data within computer-aided system. For the user interface design, we apply Adobe Flex technology to build a flexible web application, which enables rich user interaction and query answering. *Flex* is a software development kit (SDK) released by Adobe Systems for the development and deployment of cross-platform Rich Internet Applications (RIA) based on the Adobe Flash platform.

**Figure 4:**
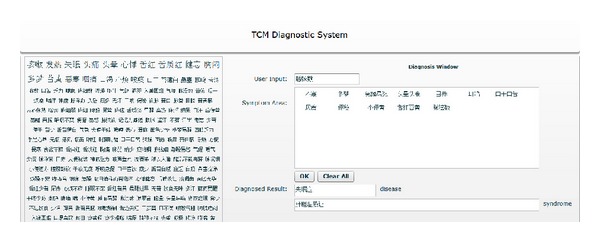
The snapshot of Ontology-oriented Diagnostic System (ODS).

In detail, our user interface is consisted of three parts: *tag cloud panel*, *input panel*, and *output panel*. Initially the user is presented with the most frequentlyoccurred symptoms in the left tag cloud panel, which provides users with an overview of all frequentlyoccurred symptoms and enable easy selection just by clicking on the word itself. The tag cloud panel guides the user in the construction of a tag cloud by means of frequency ranking. Largest texts means bigger chances to appear, smaller vice versa. The selected symptoms would be added into the input panel. Alternatively, the user can type vulgar symptoms into the input area. Entered symptoms would also be added into the input panel. Finally, all the chosen symptoms will be listed out in the right-top symptom area. The patient can fetch the diagnostic result whether he plans to do some tests on the system or have an actual requirement for medical help. The calculation for the diagnostic result is strictly behaved through our proposed methods. After the work flow, the expected disease and syndrome are listed in the output panel. A snapshot of the user interface is demonstrated as in Figure 4.

The user interaction was designed using a prototype-based interactive process, which aims to find problems and clarify the design principles as early as possible. It turns out that users prefer simplicity and vividness of our prototype, but for a diagnostic system, the accuracy of diagnostics is most important. Three metrics are imported to measure the accuracy, feasibility, and performance of the system. 

### 7.1. Accuracy

 Due to the diversity of medical domains, there are no open benchmark for testing the accuracy of diagnostic results, especially for TCM. Thus we design a metric method for accuracy: *A*–*E* value metric (*A* for “Very precise,” *B* for “precise,” *C* for “tolerable,” *D* for “erroneous,” and *E* for “wrong”).

The system was evaluated by two medical experts from CATCM, who provided 12 test cases and scored the diagnostic result in *A*–*E*. All the test cases are directly selected from the authoritative textbook of Chinese Internal Medicine, which has no direct connection with the sample database. The scoring results are listed as in Table 1, in which each domain expert gives both total accuracy (disease plus syndrome diagnosis) and syndrome accuracy, because the disease diagnosis concludes on the patient's health condition, and syndrome diagnosis matters to the accuracy of syndrome differentiation. The system can achieve good diagnostic decisions, in which the overall diagnostic accuracy (*O*) scores distributes as 67% *A*, 25% *B*, 8% *C*, and no votes for *D* and *E*. The relative score (*R*) measures the accuracy of single-syndrome matching, which distributes over 50% *A*, 42% *B*, 8% *C*, and no votes for *D* and *E*, as shown in Table 2. 

We found that most users accepted our system with positive reactions and considered the system generally useful to help medical practitioners with clinical decision making. Furthermore, we compared the diagnostic results using only minimum set extraction method and the complete ontology-oriented method, and it turned out that 6 over 12 test cases got much better results using the ontology oriented method.

### 7.2. Performance

 The performance of our system is also measured, which estimates the efficiency of our algorithms: (1) the symptom calculation of diagnostic library, (2) integration algorithm of heterogeneous data sources.

**Figure 5:**
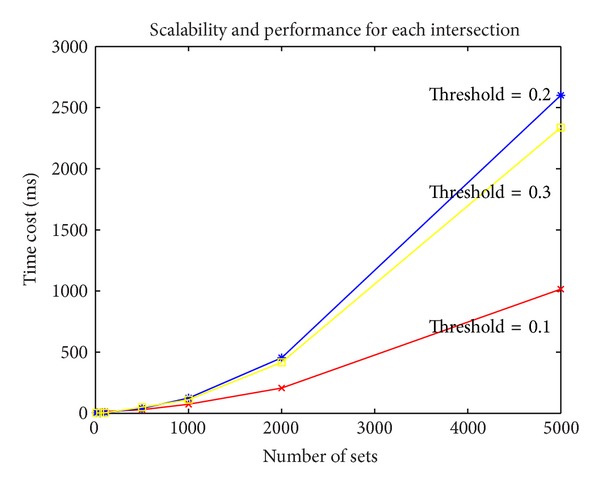
The performance curve for each intersection.

It is known that Algorithm 1 computes the *t*-threshold calculation of multisets of difficulty *δ* in *O*(*tδ*log⁡*k*log⁡*n*). From Figure 5, we can see that the computation cost of each set intersection calculation is raising by the number of sets involved, not to mention doing *t*-threshold multiple times. However, when the threshold increases, which means the condition turns to be stricter, the time cost will fall. The integration strategy checks the mathematic relation between inputs and primary result and matches extended choices by calculating similarity with low cost.

### 7.3. Scalability

 Since the practical basis of our system is mainly based on the intersection calculation of clinical cases, the generated diagnostic library could vary due to different clinical cases in terms of content and volume. Thus, the computation cost of the system is measured with the increasing number of clinical cases. The number of clinical cases will only affect the scalability of set intersection algorithm. 

 The computation cost raises fast with the increasing number of clinical cases, such that it is bearable only if it is offline preprocessing when a large number of cases and disorder concepts involve in.

## 8. Conclusion and Discussion

 In this paper, we implemented an integrated diagnostic system for Traditional Chinese Medicine, which learns diagnostic principles from prior clinical experience to provide clinical decision-making services. As an alternative source of health care, TCM is interpreted as to have intangible connections between human and nature rather than anatomical parts, which leads to complex semantic inclusion relations. We have done some researches on expressing biological facts into ontological statements, by constructing domain ontology as OWL and RDF models. Semantic technologies show great potential on querying and reasoning the knowledge of TCM [27]. The ontology assisted diagnostic system interprets the correspondence between symptoms and syndromes in an integrated method of minimum set mapping and ontology refinement, instead of static rules which are difficult to conclude. Web users could access the online user interface and fetch a diagnostic result according to the specific input symptoms.

Over 10,000 patient records are collected from curated data sources to constitute a sample database, in which attributes could be grouped into three columns: disease, syndrome, and symptoms. We run a minimum symptom set extraction algorithm for each syndrome and disease so as to generate a small featured set of symptoms for each of them to form a statistical mapping table. Since there are naming conflicts and inclusion association in TCM terms, we codify the hierarchy of syndromes and logical relations (such as class inheritance, equivalence, and inclusion) extracted from literature with the help of TCM experts into a syndrome ontology model. The finalized diagnostic result is obtained after a relation refinement process over the mapping table based on the ontology information. We process text similarity matching and ontology refinement for the input data to find the most relevant result. To verify the accuracy of our system, we invite medical experts in CATCM to provide 12 test cases and score the result of each case using a grading metric.

There are a lot to be further investigated. The key consideration of building an intelligent diagnostic agent is to understand the essence of TCM medical theories, which may consists of philosophical ideas about five phases and body, treatment principles, diagnostic methods, and so forth. The most import component of our diagnostic system is the mapping table (diagnostic library), which learns diagnostic principles by *t*-threshold intersection algorithm. The performance of the algorithm raises fast with the increasing amount of clinical cases. The performance problem may be resolved by distributing sample data into several commodity machines to reduce computation cost, probably by increment strategies.

## Figures and Tables

**Algorithm 1:**
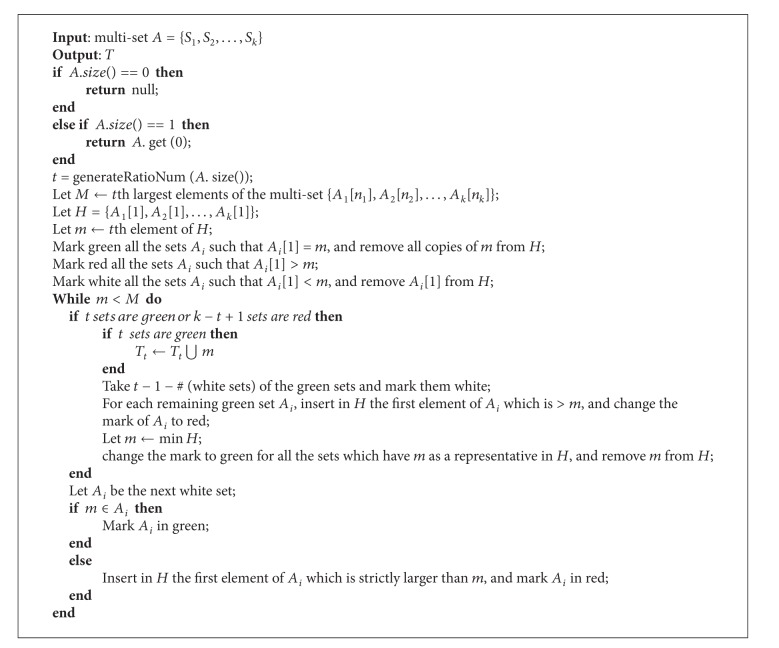
Minimum symptom set extraction.

**Table 1:** Main characteristics of *SynOnt* ontology.

Number of classes (syndromes)	391
Number of subclass axioms	483
Number of equivalent class axioms	254
Number of subsumed class axioms	122

**Table 2:** Experimental Results of ODS diagnostics (total accuracy/syndrome accuracy).

Number	Nonontology diagnosis	Ontology-oriented diagnosis
1	A/A	A/B
2	C/B	A/A
3	C/C	A/B
4	C/D	A/A
5	B/A	B/B
6	C/D	B/B
7	E/E	A/A
8	A/A	A/A
9	B/A	B/C
10	C/B	A/A
11	C/B	A/A
12	B/A	C/B
